# P-803. A 10-year Retrospective Analysis of Staphylococcus aureus Bacteremia in a Philippine Tertiary Hospital

**DOI:** 10.1093/ofid/ofae631.995

**Published:** 2025-01-29

**Authors:** Alfred Daniel M Navarro, Karl Evans R Henson, Cybele Lara R Abad

**Affiliations:** The Medical City, PASIG, National Capital Region, Philippines; The Medical City, PASIG, National Capital Region, Philippines; University of the Philippines - Philippine General Hospital, Manila, National Capital Region, Philippines

## Abstract

**Background:**

*Staphylococcus aureus* is a frequent cause of bacteremia in community and health care settings. There is limited data on *S. aureus* bacteremia (SAB) in the Philippines. This study describes the demographic profile and clinical characteristics of adult patients with SAB and their association with in-hospital mortality.

**Figure:**
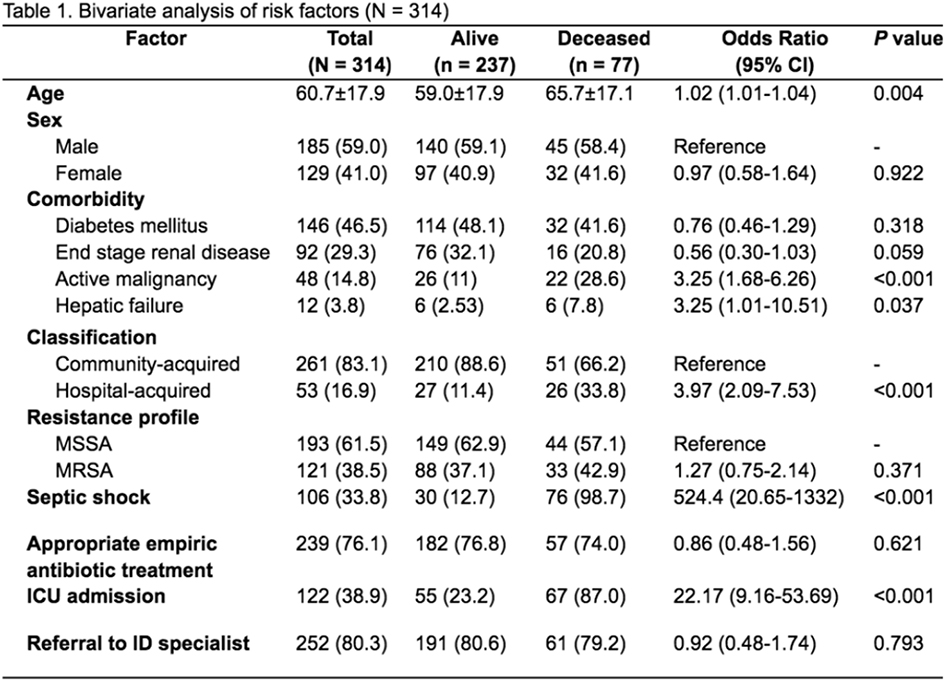

**Methods:**

Patients aged ≥ 18 years with SAB from 1/1/2013 to 12/31/2023 were reviewed for study inclusion. SAB was defined as at least one blood culture result positive for *S. aureus*. Clinical characteristics, comorbidities, sources of infection, and clinical outcomes were recorded. Stepwise regression analysis was performed using STATA 13.1.

**Figure:**
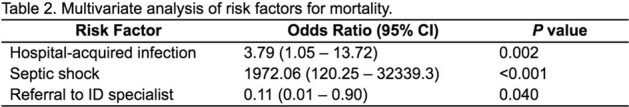

**Results:**

314 patients with SAB were included; over half were male (185/314, 59%) and majority (62.5%) had Methicillin Susceptible *Staphylococcus aureus* (MSSA) bacteremia. The most common comorbidities were diabetes mellitus (146/314, 46.5%) and end stage renal disease (92/314, 29.3%). Mortality was high at 24.5% (77/314). Deceased patients were significantly older than survivors (65.7 years vs 59.0 years, *p* = 0.004). Among deceased patients, pleuropulmonary and intravascular catheter-related infections were the most common sources of infection comprising 29/77 (37.7%) and 12/77 (15.6%), respectively. Most patients (252/314, 80.3%) were referred to infectious diseases (ID) specialists. Clinical factors significantly associated with in-hospital mortality in the bivariate analysis included older age (OR=1.02, 95% CI 1.01-1.04), active malignancy (OR=3.25, 95% CI 1.68-6.26), hepatic failure (OR=3.25, 95% CI 1.01-10.51), hospital-acquired SAB (OR=3.97, 95% CI 2.09-7.53), septic shock (OR=54.0, 95% CI 7.2-404), and ICU admission (OR=22.17, 95% CI 9.16-53.69). In the multivariate regression model, hospital-acquired SAB (OR = 3.79, 95% CI 1.05 – 13.72, *p* = 0.002) and septic shock (OR = 1972.07, 95% CI 120.25 – 32339.3, *p* = < 0.001) increased the odds of in-hospital mortality, while referral to ID was protective (OR=0.11, 95% CI 0.01 – 0.90, *p* = 0.40).

**Conclusion:**

The high mortality rate of SAB in this study is comparable to published data. Hospital-acquired SAB and the presence of septic shock significantly affect in-hospital mortality. Referral to ID specialist is associated with improved survival.

**Disclosures:**

**Karl Evans R. Henson, MD, FIDSA**, BSV Biosciences: Honoraria|Cathay Drug Company, Inc: Grant/Research Support|MSD: Honoraria|Pfizer: Advisor/Consultant|Pfizer: Honoraria

